# Reaction-diffusion in the NEURON simulator

**DOI:** 10.3389/fninf.2013.00028

**Published:** 2013-11-15

**Authors:** Robert A. McDougal, Michael L. Hines, William W. Lytton

**Affiliations:** ^1^Department of Neurobiology, Yale UniversityNew Haven, CT, USA; ^2^Department Physiology and Pharmacology, SUNY DownstateBrooklyn, NY, USA; ^3^Department of Neurology, SUNY DownstateBrooklyn, NY, USA; ^4^Kings County HospitalBrooklyn, NY, USA

**Keywords:** computational neuroscience, python, reaction-diffusion, neurodynamics, numerical integration

## Abstract

In order to support research on the role of cell biological principles (genomics, proteomics, signaling cascades and reaction dynamics) on the dynamics of neuronal response in health and disease, NEURON's Reaction-Diffusion (rxd) module in Python provides specification and simulation for these dynamics, coupled with the electrophysiological dynamics of the cell membrane. Arithmetic operations on species and parameters are overloaded, allowing arbitrary reaction formulas to be specified using Python syntax. These expressions are then transparently compiled into bytecode that uses NumPy for fast vectorized calculations. At each time step, rxd combines NEURON's integrators with SciPy's sparse linear algebra library.

## Introduction

In a quest to study the brain *in silico*, computational neuroscience has long focused on electrophysiology. This focus is partly because electrical signaling is a relatively accessible form of neuronal activity. The GENESIS [genesis-sim.org; Bower and Beeman (1998)], MOOSE [moose.ncbs.res.in], and NEURON [neuron.yale.edu; Carnevale and Hines (2006)] allow modelers to predict the collective electrical activity of networks of neurons based on their connectivity, the morphologies of the individual cells, and the distribution of ion channels.

Neurons are more than mere objects with interesting electrical properties: they are cells, and as with any cell, there is an enormous amount of chemical signaling in their interior (Blackwell, 2005; Brown et al., 2011). The cell and systems biology communities have developed many tools and methods for numerically simulating intracellular chemical dynamics. There are two broad classes of simulation types: stochastic and deterministic. Stochastic simulation is most appropriate when the number of molecules is low in a region of interest, either because the concentration is low or the region is small. Such simulations may track individual molecules in a meshless geometry as in MCell [mcell.cnl.salk.edu; Stiles and Bartol (2001)] or Smoldyn [www.smoldyn.org; Andrews et al. (2010)]. Alternatively, stochastic simulations may monitor the number of molecules by compartments and update these values via the Gillespie method (Gillespie, 1976) or related algorithms. When the dynamics are insensitive to changes in a small number of molecules, deterministic simulation may be employed. In this case, the dynamics may be expressed in terms of a partial differential equation (PDE) which may be solved by a variety of algorithms, including finite elements and finite volumes. Finite volumes turns the PDE problem into a large system of ordinary differential equations, which may be then solved using a variety of techniques, some implicit some explicit. The Virtual Cell [nrcam.uchc.edu; Loew and Schaff (2001)] and STEPS [steps.sourceforge.net; Wils and De Schutter (2009)] support both stochastic and deterministic simulation. In some models, there may be more possible states than expressed states, for example when a molecule has a large number of binding sites. Rule based strategies, such as are used with BioNetGen [bionetgen.org; Faeder et al. (2009)], allow the efficient simulation of such models, with minimal performance penalty for unexpressed states.

Electrical and chemical signaling not only coexist: they are intimately involved with one another (Blackwell, 2005). Membrane potential variations, the basis of electrical signals, are created and maintained through the movement of ions through ion channels and pumps. These ion channels and pumps are modulated by chemical factors both inside and outside the cell. Meanwhile, these same chemical modulators factors are themselves created or admitted by membrane processes that are responsive to electrical potential and to other modulators (De Schutter, 2008). Experimental neuroscience is developing new methods to probe chemical dynamics and electrophysiology simultaneously. These new techniques provide us with new data to constrain our computational models, and new ability to evaluate our models by making testable predictions. Parallel innovation in both experimental and simulation neurotechnologies move us toward connecting these previously distinct domains of research. This is effected by development of multiscale models which enable us to connect pharmacological causes (chemical treatments) to clinical effects—treatments of neurological diseases manifesting at neuronal, neuronal network, and higher scales (Lytton, 1997; Ferrante et al., 2008).

NEURON and GENESIS have long been sufficiently general that they can support such multiscale models. In NEURON's case, arbitrary reaction dynamics can be specified in NMODL with diffusion via NMODL's LONGITUDINAL_DIFFUSION statement. For GENESIS, the Chemesis tool [krasnow1.gmu.edu/CENlab/software.html] provides similar functionality. Unfortunately, this previous NEURON approach is very demanding of the modeler who must (1) write and debug NMODL, a compiled language; (2) explicitly handle any subcompartment discretization including fluxes between subcompartments, and (3) adjust concentrations due to membrane fluxes. This last task requires computing the correct surface-to-volume ratio, which is complicated due to the fact that NMODL can only directly access the diameter at the midpoint of the segment. Stochastic changes are possible, but only if the corresponding algorithm is explicitly embedded in the NMODL.

To better address the needs of multiscale modeling, we have now re-implemented the NEURON simulator's approach to the specification of reaction-diffusion models. From this, the scale of molecular interaction can be coupled to the scales of single-neuron and network modeling that has been NEURON's focus (Carnevale and Hines, 2006). This rxd extension introduces support for multiple regions, thus supporting certain classes of segment subcompartments. Users specify regions in geometric terms and the extension uses this data and the full morphology information to handle fluxes and compute accurate surface areas and volumes. In this way, the specification of geometry has been separated from the specification of dynamics. Likewise, the required skill levels and risk of user error have been reduced. We designed our model specification format in a way that is independent of the dimensionality of the discretization and of integration strategy. This extension—distributed with NEURON as the Python module neuron.rxd—may be freely used and extended under the terms of the GPL license, version 2.

The development version includes experimental support for deterministic three-dimensional simulation, and we are actively working on stochastic support. For this paper, we focus on using this extension in a one-dimensional deterministic context, support for which was first introduced in NEURON 7.3.

Certain key parts of the NEURON reaction-diffusion extension were necessarily written in C/C++ in order to obtain maximal efficiency for inner loops. However, most of the code, including all of the interface code, was written in Python (Davison et al., 2009). This choice was facilitated by the fact that NEURON already utilizes Python as one of two supported interpreted languages for controlling NEURON models (Hines et al., 2009). The ctypes module provided ready access to NEURON's otherwise-unexposed internal methods, including the newly written reaction-diffusion support code. In the development version, we have begun to use the Cython compiler to accelerate selected Python code, a process which is continuing.

## Examples

To place this work in specific contexts, we utilize two examples: the first is a simple phenomenological model of wave propagation, while the second compares the NMODL and rxd ways to handle ion accumulation and diffusion in a model taken from the literature.

### Wave propagation

**Figure 1:**
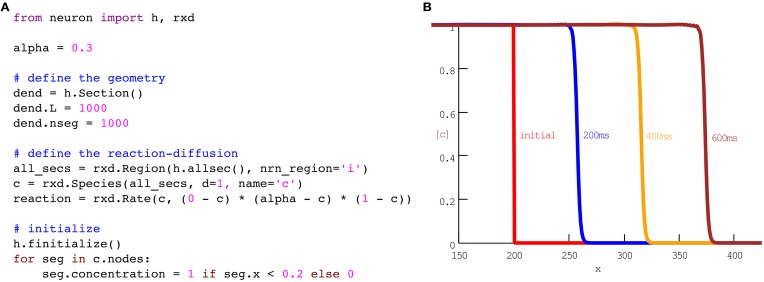
A phenomenological model of wave propagation, implemented in eleven coding lines of Python **(A)**. **(B)** Plots of concentration vs space at times as indicated. No additional code is needed for the run or plotting; this panel is the result of running the code in **(A)** with the CVode method using NEURON's standard Run Control panel. The plot with labels was generated with NEURON's Shape Plot GUI tool. The 600 ms simulation ran in 1.06 s on our test machine.

Just as action potentials are propagating waves of electrical activity, some chemical signaling occurs in the form of concentration waves. For example, multiple groups have sought to model calcium waves in pyramidal cells (Coombes et al., 2004; Peercy, 2008). The full models involve multiple species and channels. For simplicity, we begin with a single species and a single reaction that together exhibit key features of the wave: (1) calcium diffuses, (2) at low concentrations, calcium is cleared, while (3) above a threshold, calcium will tend to an asymptotic value. One example of a rate of change that satisfies (2) and (3) is (0 − *c*)(α − *c*)(1 − *c*). For concentrations *c* between 0 and α, the rate is negative, so concentration will decrease. Conversely, between α and 1, the rate is positive and concentration will increase. When combined with diffusion, the corresponding equation is known as the scalar bistable equation, which is a problem that has been studied extensively in its own right (Fife, 1979). In our framework, this model may be implemented in eleven lines of code as shown in Figure 1.

### Accumulation and diffusion: NMODL vs rxd

We now consider a more typical reaction-diffusion problem with a branched geometry and interaction with ion channels. Fleidervish et al. (2010) models the influx and diffusion of sodium ions in response to action potentials in a stylized pyramidal neuron. These changes modulate ion channel dynamics by altering the reversal potential, and in other models, channel gating may also be sensitive to ion concentration (for example, models with calcium gated potassium channels).

**Figure 2:**
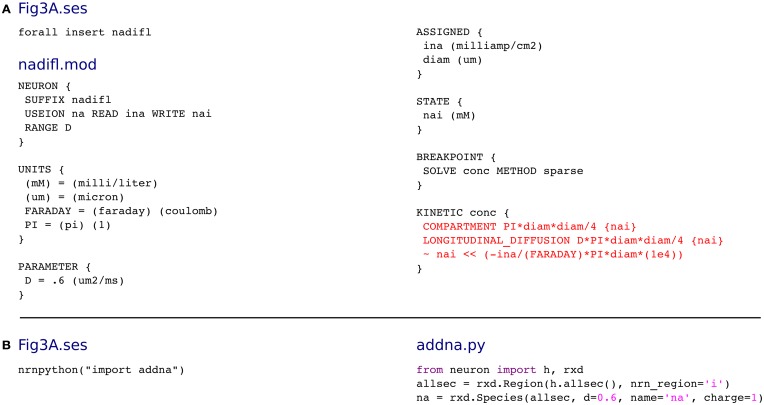
**(A)** The traditional way of modeling sodium increase due to ion channel activity and diffusion in NEURON, excerpted from the model of Fleidervish et al. (2010). The core of the calculation (red) mixes the geometry and the modeling. To make a fair comparison, Figure 3A.ses has been modified from the original version which used four lines of automatically generated code to insert the nadifl mechanism. **(B)** In the rxd approach, the code is shorter and clearer as less connectivity code is needed and as rxd automatically handles ion influx and the effects of geometry on diffusion.

The traditional way to express these sodium kinetics is shown in Figure 2A, excerpted from the ModelDB entry [senselab.med.yale.edu/modeldb/ShowModel.asp?model=136715] for Fleidervish et al. (2010). The three red lines, in order: (1) define the volume per unit length, (2) define the diffusion rate, here the diffusion constant times the cross-sectional area, and (3) describe the change in ionic mass due to membrane flux, which is proportional to the current times the surface area. The geometry is mixed in with the description of the dynamics. The rest of the file provides the information necessary for NEURON to use this mechanism. The same dynamics can be specified with far fewer lines using the rxd module, as shown in Figure 2B. The resulting 690 compartment model runs to 3000 ms in 1.49 s on our test computer.

## Organizing reaction-diffusion simulations

NEURON is a simulation environment that has been built up over the past several decades through accretion, with the addition of multiple integrators (e.g., CVODES, LSODA), a graphical environment (InterViews), an interpreted language by Kernighan and Pike (HOC) (Kernighan and Pike, 1984), a compiled kinetics definition scheme from the National Biomedical Simulation Resource (NMODL) (Hines and Carnevale, 2000), morphology tools for Eutectics and Neurolucida microscopy programs, and several others (Gardner et al., 2003). One of the challenges of working in this complex, multitool environment is the need for coordination between and across the pieces. The recent addition of Python to this suite is highly advantageous since it gives immediate access to a number of immediately useful tools: NumPy, SciPy, Matplotlib, etc. Additionally, as we will show here, Python can provide “glue” across the different internal components. To do this, Python must be coordinated with the other 2 languages, with the integrators, and with the GUI.

A user working in Python enables the reaction-diffusion extension via import rxd. Because the two coexisting interpreted languages, Python and HOC, can freely call each other's procedures and access each other's objects, there was no need to develop a separate interface for the HOC language. This feature was particularly valuable in development of the experimental rxd graphical user interface (GUI), which is written using the existing NEURON interface to InterViews instead of a Python GUI library to provide a consistent look-and-feel familiar to the NEURON user. Similarly, additional simulation scripting of this Python-based product can be done in either Python or HOC. For the remainder of this text, we use Python examples.

We organized the simulation-scripting interface through a set of questions that step the users through a logical sequence to define 3 major aspects of structure and function required for performing this type of simulation. (1) Where do the dynamics occur? This is the primary structural question—we need to define the spaces that will be involved. Generally these include at least cytoplasm, but often other internal compartments will also be involved. Limited support for diffusion in the extracellular Frankenhaeuser–Hodgkin space is provided, and we intend to further support extracellular diffusion in the future. (2) Who are the actors? All diffusing substances must be identified; these provide the state variables for the system. (3) What are the reactions? We must determine which of the substances reach with which other substances.

Although this sequence appears straightforward, there are known complexities that we do not yet handle. A consistent difficulty with biological reaction-diffusion is the following: how best to identify a substance which can undergo a series of minor alterations through phosphorylations or through calcium or other cofactor bindings (Keller et al., 2008; Pepke et al., 2010; Lisman et al., 2012). For example, the combinatorics of *n* phosphorylation sites allows these modifications to result in a single substance, typically an enzyme, having the possibility to take on 2^*n*^ forms, in addition to the isoforms that arise based on alternative polypeptide subunit composition (Kelly et al., 2007). For such cases, it is parsimonious to consider the forms (and isoforms) as simply being variations on the same substance, saving us from having to identify thousands of different variants with their associated inter-variant reaction rates. Note that these variants may affects rates of reactions with other substances (also multiform), so that the current identity of an ensemble of forms plays a role in determining reactions as well.

### Where do the dynamics occur?—the domains

For traditional neurophysiological simulations, the only areas of interest were the plasma membrane and the 2 regions immediately adjacent to the membrane on either side. The membrane provided the capacitance, and provides pores that are handled as resistors and rheostats. The cytoplasmic and extracellular regions directly adjacent to the membrane provide the Nernst potentials, modeled as batteries in series with these rheostats and resistors. By contrast, computational cell biology both takes us away from the membrane littoral, into the depths of the cytoplasm. This modeling also plumbs a variety of subcellular compartments (endoplasmic reticulum, mitochondria, nucleus, etc.) that play major roles in intracellular signaling, as well as into a large number of substances and reactions that are the underpinnings of cell structural support, metabolism and waste disposal, but may play a secondary role in physiological neural signaling or in neuropathology.

A domain is specified through the creation of a Region object. The dendritic (or axonal) tree in NEURON is divided up into unbranched sections which end at branches. The single required parameter for a Reaction constructor is a list of these sections describing the morphology which contains this Region. If the Region corresponds to NEURON's standard cell-inside or cell-outside, this is specified with the nrn_region keyword argument. Finally, geometries must be specified. NEURON ships with several standard geometries including shells, outer membrane definition, and fractional volumes. Users are free to implement their own using custom geometry objects within our interface. Other geometries will come from electron microscopic tracings.

For example, the following statement defines a border as the shell between 90 and 100% of the dendritic radius across all sections:


border = rxd.Region(h.allsec(),
         nrn_region= ‘i’,
         geometry=rxd.Shell(0.9, 1.0))


The optional choice of ‘i’ for nrn_region sets up the region to correspond to NEURON's inside, the region, which affects and is affected by NMODL file compiled reaction and channel dynamics. In a one-dimensional branching model, the exact geometry of intracellular compartments has no effect on the dynamics as long as the length, volume, and areas are correct. For example, if the endoplasmic reticulum (ER) occupies the fraction fe (0 ≤ fe ≤ 1) of the cross-sectional area (and hence fe of the volume), one may model that region as:


er = rxd.Region(h.allsec(),
                geometry=FractionalVolume
                (volume_fraction=fe))


No nrn_region is specified as the ER concentrations do not directly affect the activity of ion channels on the plasma membrane.

**Figure 3:**
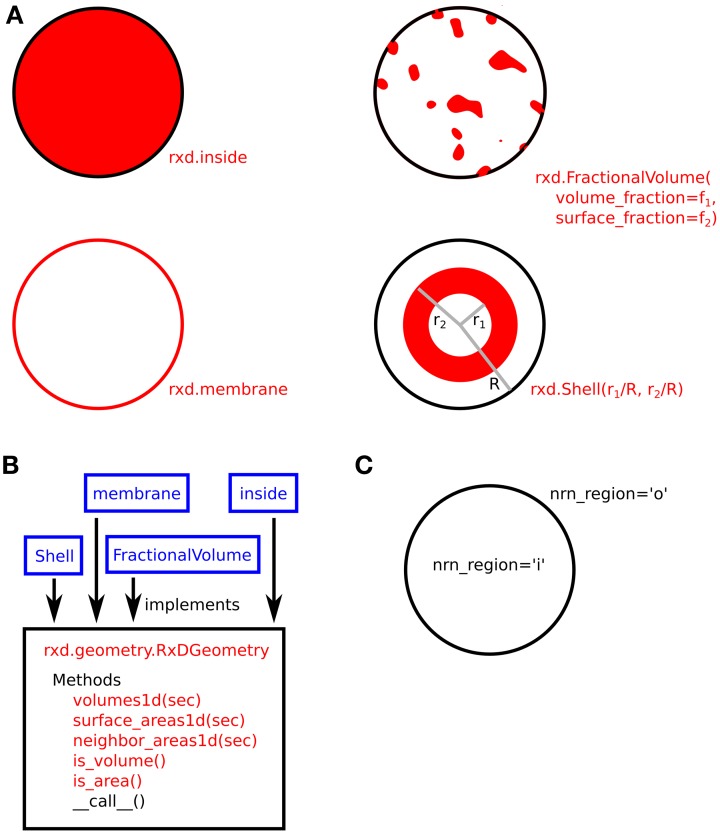
**(A)** Four examples of compartment geometry supported by rxd. In NEURON, the dendritic cross-section is approximated as a circle. The red colored areas correspond to the portion of the cross-section described by the given geometry. FractionalVolume is the most general and is intended for cases where the region occupies a complexly-shaped subset of the cross-section. **(B)** These classes (blue) implement the rxd.geometry.RxDGeometry interface; users may extend rxd with new geometries by writing new classes that implement the same interface. **(C)** Named Species (e.g., ca) living in a Region with nrn_region=‘i’ map to concentrations in NMODL's inside region (e.g., cai). Similarly, ‘o’ maps to NMODL's outside region.

The use of Region arguments to specify the geometric shape and NMODL location of compartments is illustrated in Figure 3.

### Who are the actors?

The actors, or reactants, in a reaction-diffusion module may either be diffusible rxd.Species, or fixed objects (e.g., channels and pumps). Fixed objects are generally defined by one or more rxd.State objects, as in the gating variables (*m, h, n*) in the classic Hodgkin–Huxley equations. These states are conceptually distinct from a species that takes on a variety of phosphorylation ‘states’ discussed above.

The Species constructor has only one mandatory argument: the Region where it will exist. It also accepts additional optional attributes, such as: a name, diffusion rate, charge, and initial distribution of concentration. All of these may be set or initialized in the constructor. A name argument is necessary only when a Species will also be acted upon by non-rxd mechanisms, e.g., an ion that will be sourced via a membrane ion channel and will play a role in determining a Nernst potential. The need for both a name and a Python handle is the only case where the complexities of coordination between the different components is directly exposed at the user interface.

When a Species has a global NEURON name and is expressed on a Region where nrn_region=‘i’ or nrn_region=‘o’, then the concentrations are fully accessible by HOC, the GUI (e.g., Channel Builder), and by NMODL. For any such Species that pass through the cell membrane, the charge of an individual ion must be specified, in order to connect flux to current. In addition, an initial concentration distribution is needed. This may be expressed as a constant or as a function that depends on each rxd.Node where the Species is expressed. Alternatively, the distribution may be omitted, in which case it will be taken from its definition within NEURON. If both Python and NMODL/HOC define different distributions, the Python initialization takes precedence.

For example, to define the Species ca (Ca^2+^) with a diffusion rate of 0.3 μm^2^/ms and a uniform initial concentration of 5 μM = 0.0005 mM on the regions cyt and er, we use:


ca = rxd.Species([cyt, er], d=0.3,
       name= ‘ca’, charge=2, initial=0.0005)


The first ca is the Python handle for the Species and is used to adjust properties (e.g., to set the concentration in the er: ca[er].concentration=0.0009) and for defining reaction schema and rate expressions. We again note the name=‘ca’ argument, ideally set identically to the Python variable name as here to prevent future confusion, couples this Species into mechanisms established in NMODL or HOC.

### How do they interact?—the reactions

**Figure 4:**
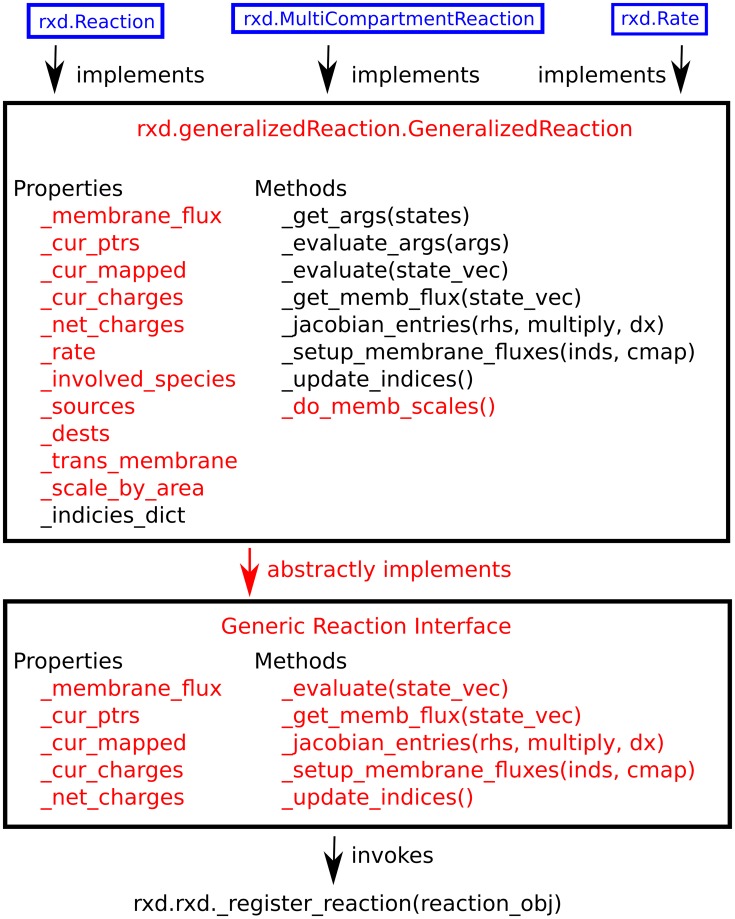
**Hierarchy of reaction classes**. Red denotes abstract methods or properties, while black text indicates implemented ones. New schemes for specifying reaction kinetics may be implemented using the generic reaction interface, GeneralizedReaction, or any of the three reaction classes provided with rxd (blue).

Within the context of reaction-diffusion the concentrations of Species change through interactions, generally stoichiometric, with other Species. We provide two tools for specifying these primary interactions: rxd.Rate, and rxd.Reaction. The rxd.MultiCompartmentReaction class manages situations where reactions are occurring across different regions. All three tools are subclasses of rxd.GeneralizedReaction. Utilizing the flexibility of Python and the strength of inheritance, a user can readily implement a custom subclass for additional needs that we have not anticipated. Because the underlying numerical algorithms interact with tools through the GeneralizedReaction interface, such a custom reaction subclass will have access to the full array of NEURON components. This hierarchy is illustrated in Figure 4. These mechanisms localize by default to any and all Regions wherein all of their state variables are present. It is possible to override this by setting optional keyword argument regions.

Each of the three reaction classes accepts reaction rates specified as arbitrary algebraic combinations of numbers, State, Species, and Parameter objects. For example, a rate of 2+ca is interpreted as two more than the local ca concentration at a given time step. A variety of functions (e.g., log, sin, etc.) are also supported; these are provided in the rxdmath submodule. Support for other functions may also be added by the user at runtime. The Reaction and MultiCompartmentReaction constructors accept three or four positional arguments: reactants, products, forward reaction rate, and an optional backward reaction rate. The reactants and products are specified as molecular combinations (stoichiometric sums of positive integer multiples of a Species).

For example, 2 * hydrogen + oxygen ↔ water. The full reaction with forward rate kf and reverse rate kb would be expressed as


r = rxd.Reaction(2 * hydrogen + oxygen,
      water, kf, kb)


Both Reaction and MultiCompartmentReaction assume mass-action kinetics by default, meaning that the stoichiometry coefficients are implicit. Therefore, the rate of change of hydrogen here will be -2*kf*hydrogen^2^*oxygen+2*kb*water mM/ms. In a stoichiometric context, a reaction is not an equality so factoring does not work: 2*hydrogen+oxygen↔water differs from 4*hydrogen+2*oxygen↔2*water. An alternative means of expression uses the keyword argument mass_action=False. In this case, kf and kb will be interpreted as the full forward and reverse rates. In that case, the rate of change would be kb-kf mM/ms for oxygen, and 2*(kb-kf) for hydrogen.

MultiCompartmentReaction objects, reactions spanning multiple compartments, differ from Reaction objects in three key ways: (1) Region objects for all the state variables must be explicitly specified; i.e., instead of writing ca, one must write ca[cyt], ca[er], etc. This specification allows unambiguous interpretation of the reaction scheme and reaction rate. (2) The mandatory argument membrane= specifies the two-dimensional region that separates the two compartments; this often corresponds to a physical membrane such as the cell membrane or the ER membrane, but could also refer to a conceptual boundary between two adjacent shells. By default, the local rates are scaled by membrane area; this is important for pumps and channels lying on the membrane, but can be disabled by setting the optional keyword argument, scale_by_area=False. (3) Since the rates are proportional to membrane area and since the volumes of different compartments may differ, the units for rates with mass_action=False are in molecules/μm^2^/ms, with appropriate corrections for the default mass action case.

Some MultiCompartmentReaction fluxes induce electrical potential changes across the associated membrane. In some cases a molecule is modeled as if it crosses the boundary in two stages, first binding to a species on one side of the membrane, and then unbinding on the other side after a conformational change. In order to avoid double counting of flux in such a case, the user is required to explicitly say which MultiCompartmentReaction objects induce current. This is done by the MultiCompartmentReaction keyword argument membrane_flux=True, which defines that the movement of the ion across the associated membrane will induce a change in that membrane's potential (typically, but not necessarily, the plasma membrane). The total magnitude of potential change is then calculated from the number of molecules of each species moving across the membrane and the charge of each species.

A Rate is the more general scheme. It is therefore the simplest, yet the one that requires the most complete definition by the modeler. Rate takes two arguments: a variable to change and an expression of the rate of change. This expression is added directly to the right-hand-side of the differential equation for the State or Species. For a unitless State variable the expression are in units of 1 ms, while for Species, the units are in mM/ms (note that for consistency with the rest of NEURON we use units of mM rather than the μM that is more typically used in computational systems biology).

For example, to describe degradation of ip3 according to Michaelis-Menten dynamics, we write:


ip3_degradation = rxd.Rate(ip3,
                    -ip3/(kd + ip3))


where kd is the dissociation constant.

### Tutorial

Additional usage information and examples are available on the NEURON reaction-diffusion tutorial at neuron.yale.edu/neuron/static/docs/rxd/index.html.

## Implementation details

### Initialization and run-time

A key consideration in the development of the rxd extension is that the added functionality should not introduce a performance penalty on non-reaction-diffusion simulations. To prevent this, we added hooks for connections in the form of function pointers to the NEURON core. If these pointers are unset, then no function is called and the only performance penalty is a single if statement per hook invocation. With from neuron import rxd, the extension registers 1. an initializer (h.FinitializeHandler) to catch initialization events; 2. a transfer agent (CVode.extra_scatter_gather) for transferring concentration data after an advance; 3. an additional solver (nonvint_block_handler) for the insertion and solving of the reaction-diffusion state dynamics. These individual calls remain light-weight until and unless a particular model is placed in a region.

The user then defines morphology and Region, Species, Reaction objects. When instantiated, each of the main reaction-diffusion classes besides Region registers themselves with a global list. For Region, there is no significant additional computation at instantiation. A Species, by contrast, at instantiation reads the connectivity information and allocates memory for each node. Reactions convert the rate formulas into vectorized expressions at instantiation. At the beginning of run-time, the model is initialized (h.finitialize() called) to initialize concentrations and construct the diffusion matrix.

### Numerical integrations

**Figure 5:**
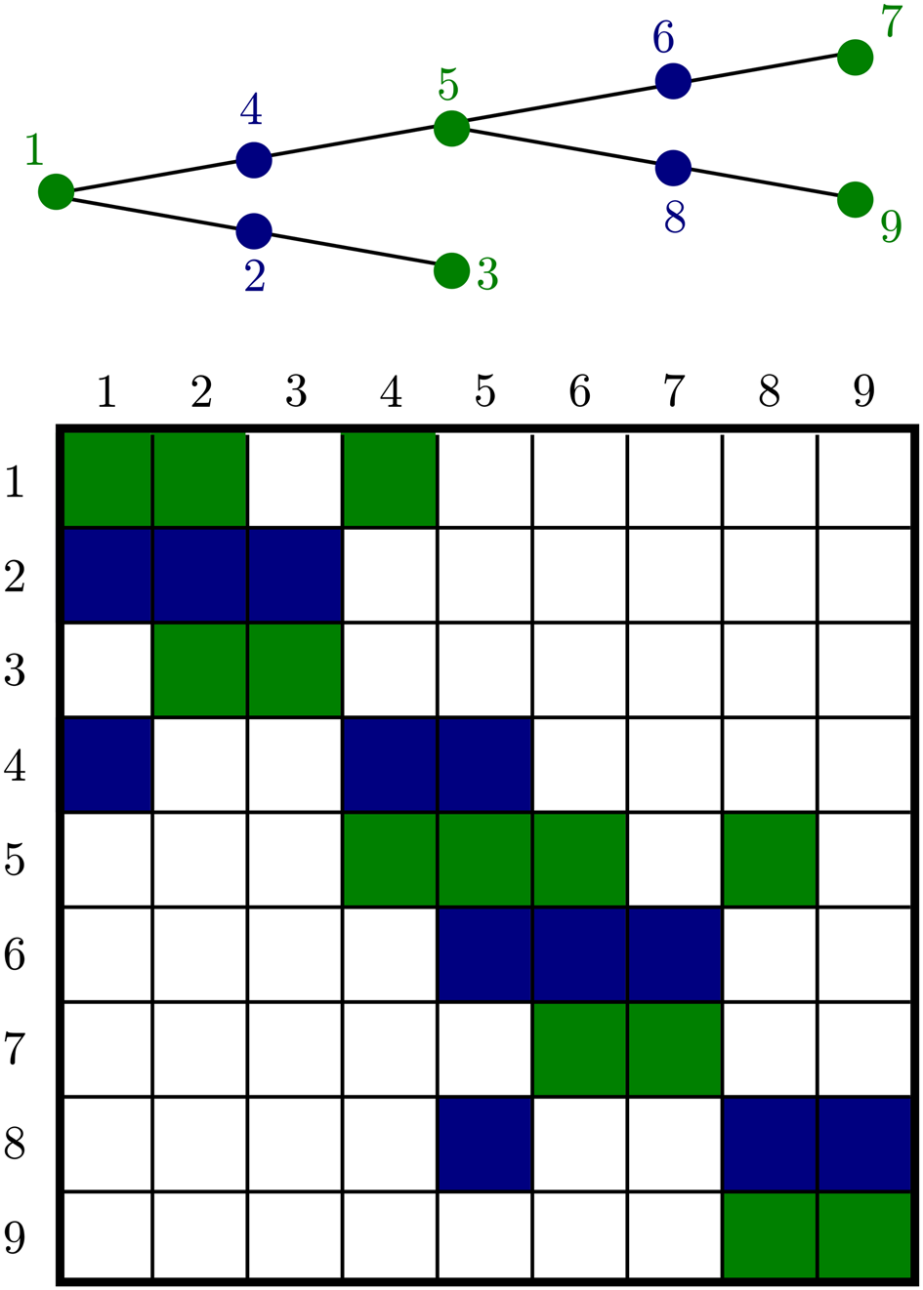
**A branched geometry (top) and the structure of the corresponding tree matrix (bottom)**. Nonzero matrix entries are colored either green or blue depending on the color of the node corresponding to the row. The green nodes at the ends of each segment are algebraic conservation nodes and so the corresponding green rows each sum to 0. The matrix need not be symmetric; *a*_*ij*_ is not in general equal to *a*_*ji*_ because the entries depend on the volume of compartments *i* and *j*, respectively.

Although general diffusion has a fairly complex Jacobian structure, 1-dimensional diffusion in a dendritic tree gives a matrix with a special structure, Figure 5. This is an important advantage for handling diffusion in neurons, on dendritic trees extending for up to 1 mm, and axons which may extend more than a meter. This algorithm allows solution of matrix equations in O(*n*) time. We factored the treesolver algorithm Hines (1984) out of NEURON's longitudinal_diffusion mechanism code into the C function nrn_tree_solve, allowing us to exploit our knowledge of the structure instead of resorting to a general purpose routine.

By default, NEURON uses implicit Euler to integrate the equations underlying electrophysiology models, an algorithm that provides certain guarantees about numerical stability. The challenge with implicit Euler is that it requires solving an algebraic system of equations which requires inverting a matrix, which is an O(*n*^3^) operation in general. We follow the prior NEURON conventions of decoupling the reaction contributions to the Jacobian from the diffusion contributions by performing separate matrix inversions for each. This results in an approximation of the solution, which reduces the convergence rate of the simulation, but allows us to use optimized techniques for each part of the problem. As the Jacobian contributions from the reactions are factored out and localized to a spatial location, these blocks can be solved in parallel as well.

Reactions are inherently localized. That is, the reaction matrix is, up to permutation of rows and columns, a block diagonal matrix, where each block corresponds to a specific spatial location. These matrix equations may be solved using LU decompositions in O(*m*^3^) operations, where *m* is the size of an individual block. When using the CVODE integrator, we further optimize this calculation by computing a sparse LU decomposition with scipy.sparse.linalg.factorized, and only update the decomposition whenever CVODE requests that the Jacobian be updated, typically once every ten time steps or so. The rxd module is the first part of NEURON to take advantage of this optimization; the rest of NEURON calculates the Jacobian at every time step.

In the fixed-step algorithm, at each implicit Euler advance, the currents are read and the reaction rates are computed. For both the fixed step and variable step methods, the combined effects of diffusion and reaction are approximated by doing the diffusion first and then the reactions, in analogy with the NEURON longitudinal_diffusion mechanism. This fixed-step error introduced by this approximation goes to 0 as dt goes to 0. The variable step method only requires an approximation to the Jacobian solution, so to the extent that the error is non-zero, it only serves to reduce the variable step size, but not to introduce error.

For fixed step, the Reaction contributions to the Jacobian are calculated at each iteration. For the variable step method, we gain performance by only updating the Jacobian when the CVODE integrator requests that it be updated.

At the end of each time step, the rxd computed states are scattered to the non-rxd parts of NEURON so they can affect ion channel kinetics and be plotted.

### Development of the formula-specification format

NEURON models were traditionally written in two parts: a description of channel dynamics written in NMODL, and control and analysis code written in HOC. This is a powerful approach as it allows the specification of arbitrary dynamics at machine speed due to NMODL's support for embedding C code, but it simultaneously introduces a barrier for entry as it required users to learn at least two programming languages, one of which (NMODL) requires a separate compilation step.

For this reason, a ChannelBuilder tool was introduced, which allowed the specification of certain classes of ion channels without NMODL: For each channel, ChannelBuilder is patterned after the KSChan class from Catacomb2 (Cannon et al., 2003), giving precompiled, parameterized code for common channel dynamics. In theory, end users can create KSChan instances themselves, but in practice, manual creation appears unused as none of the 6 models in ModelDB containing the word “KSChan” directly create a KSChan instance. Instead, it appears that those who use this tool save the state of the entire tool and use that to create KSChan instances as needed.

We improved on the ChannelBuilder paradigm when developing our reaction specifications. With ChannelBuilder, the GUI constructs KSChan objects. We provide a GUI that constructs rxd objects. Unlike the KSChan objects, these rxd objects are (1) more general as they can express arbitrary kinetics, and (2) explicitly name all parameters that are used. We discuss our approach for achieving the first improvement below, which immediately gave us the second benefit as well.

Early in our prototyping of this new tool, we specified each reaction as a Python def. Although this was the most powerful approach, we determined that it was too demanding of the user. For example, either the code must be called once per each spatial locations, or the code must be vectorized in some way. The first approach is impractically slow in Python, while the second approach requires the user to know what is automatically vectorizable and how to manually vectorize the rest.

For our next version, we defined the reactions via strings, which were then compiled into either HOC byte-code or NumPy-vectorized Python byte-code. Although these approaches were faster than looping over Python functions, the use of strings presented a new problem: referencing state variables. One option was to mandate defining a name for all state variables, but this approach was rejected because of the confusion that could result if the internalized Python name or names did not match the rxd-defined name. Another approach was to get variable names from Python or HOC, but this introduced ambiguity due to scoping: for example, is the object that matters the one assigned to the variable name at reaction specification or at run-time? To check at run-time potentially would require checking every time-step, which would introduce unacceptable overhead.

A successful solution involved the use of Python's operator overloading ability. We developed a general class called Arithmeticed, where all arithmetic operators were overloaded. These operators build a structure but do not actually evaluate any numbers. Arithmetic between Arithmeticed objects and numbers is done by first converting numbers to the Arithmeticed class. We then implemented most of the functions from the math library as functions on Arithmeticed objects. As with the arithmetic operators, these functions build algebraic structure expressing the function and the object the function works on, but do not immediately perform any math calculation.

Each Species, like every other Arithmeticed object, supports a _semi_compile method which generates a string representation of the object by supplying the unique part and recursively calling its children. This method is called by Arithmeticed._compile which calls _semi_compile on the entire expression and then evals the string to generate a Python byte-code-compiled form, typically using NumPy for vectorized calculations.

The operator overloading strategy has the advantage of allowing Python to handle the parsing, thereby eliminating parsing as a potential source of error. It also allows the same code to handle rate equations and reactants/products specification. Valid reactant specifications also do not contain functions, non-integer numbers, or operations other than addition and multiplication by an integer.

### NumPy and vector array sharing

Because we use both NEURON and Python libraries for calculations, we must seamlessly share data between the two environments. On the Python side, SciPy libraries work well with NumPy arrays; while on the NEURON side, the calculations use either C arrays or HOC Vector objects. At the user level, data copying between HOC vectors and NumPy arrays is accomplished with to_python and from_python in HOC. However, this copying is inefficient and must be avoided in any run-time calculations. We therefore extended NEURON's Vector class with a method as_numpy which uses numpy.frombuffer to return a NumPy array that accesses the same memory and hence has the same values as the Vector. This class is now available at the user level as well: data storage can be shared using numpyarray = vec.as_numpy().

For the reverse operation of getting a HOC reference to a NumPy array, we added the function neuron.numpy_element_ref(numpy_array, index). These HOC references may then be passed to NMODL as double* or used with NEURON tools for recording or plotting variables.

### Neuron's internal scatter/gather

The NEURON simulator's fundamental conceptual unit is the section, an unbranched cable ending in a bifurcation. These sections are divided into segments, each a localized region of a neuron that is electrically represented by the standard parallel-conductance model, and is conductance-linked to neighboring segments on the same or neighboring sections. Each segment has many properties: diameter, membrane potential, chemical concentrations, resistivity, etc. The internal representation reflects this conceptualization, with all the properties for a given segment stored in one data structure. Unfortunately, this memory structure does not work well with general purpose solvers which typically expect a contiguous state vector. NEURON gets around this problem with gather operations that collect the states into a contiguous vector and scatter operations that distribute the results to the segment-based memory structures at each time step.

We replicated this paradigm for reaction-diffusion integration for those species and regions that correspond to states needed by the rest of NEURON. We could have transferred the data by using dot notation (ı.e. seg.state), but this would require looking up the memory address for each state of each segment at each time step, inefficient since these memory addresses rarely change. In C, we would store these addresses in an array of pointers, but Python does not have a concept of pointer. NEURON provides a class to Python that encapsulates pointers to state variables. We initially looped through a Python vector of NEURON pointer objects and a NumPy array of states to transfer the data, but we found that was still too slow. We thus implemented a new NEURON class, PtrVector. Instances of this class store lists of NEURON pointers and are able to scatter/gather data to/from the pointer locations and an arbitrary NEURON vector.

The pointers were thus set to point to the segment-based memory locations. To scatter states, we use an intermediate NEURON vector. We interpret this vector as a NumPy array using the techniques described above, and use NumPy's indexing to copy data from the full state vector into this array. We then invoke the PtrVector's scatter method. The much rarer gather operation, needed only when a state value is changed in a way inconsistent with the differential equations, is performed in the reverse order.

The use of the intermediate vector incurs a small performance penalty, however it provides a natural place to combine state values in the general case where reaction-diffusion nodes do not correspond directly to segment, such as in the 3D simulations which we are now beginning to implement. If the memory addresses have changed, then the internal variable structure_change_count will be changed. Therefore, before each advance we check this variable and update the pointers if necessary.

### Increasing modularity and extensibility

In its over three decades of development, NEURON has acquired a great deal of functionality to support both numerics and analysis. In the process, it has also acquired great complexity. As we developed and continue to develop the reaction-diffusion extension, we located a number of places where the new code had to connect with the existing core. Instead of solving this problem once for rxd and having to resolve it for future NEURON enhancements, we have implemented general interfaces that we anticipate will facilitate future development.

#### Integration

NEURON supports a variety of integration strategies, as different methods are appropriate for different situations (Lytton and Hines, 2005). The two broad categories are fixed step methods and variable step methods. The fixed step solver can be set to run with first-, or second-order convergence depending on the relative importance of error convergence and stability. The most general single threaded variable step solver uses IDA from the SUNDIALS: suite of non-linear differential/algebraic equation solvers (Hindmarsh et al., 2005) to solve differential-algebraic equations such as arise from neurons that are coupled together by electrical circuits. When there are no algebraic equations present, the variable step variable order solver CVODE is a better choice as it is generally faster than IDA, due to its support for the treesolver method discussed above.

Additional complexity arises from the fact that these methods may be executed in a single thread or in multiple threads. The fixed step and variable step methods are fundamentally different in that the fixed step algorithm requires voltage states to be calculated half a time step away from the non-voltage states while the variable step methods treat all the states as belonging to one vector. Some of these integrators also have cache-efficient variants, which increase performance at the cost of decreased robustness and transient increases in memory usage. To deal with this diversity of cases, NEURON contains conceptual repetitions of many aspects of the integrators spread throughout the code base, but there was previously no central interface which could allow a single extension to work with these multiple cases.

In producing the rxd extension, we realized that both this and subsequent extensions would benefit from such an interface, which we called nonvint_block_supervisor (“nonvint” standing for non-voltage-integration). The handler for this is a Python module, so it is trivially able to connect to other Python modules, like the rxd extension. On the NEURON side, it had to connect in a way that did not introduce any Python dependencies, and had to allow the rapid sharing of data between the NEURON core and Python extensions.

This was accomplished by introducing a function callback in the NEURON code which is filled on import of the nonvint_block_supervisor via ctypes. Along with array pointers and array size, the callback arguments include a command type which specifies which of the 11 locations in the internal NEURON fixed and variable step methods need equation processing mediated by the nonvint_block_supervisor.

On the C side, we defined different macros for each of the 11 types of functions that the supervisor needed to be able to do: setup, initialize, current, conductance, fixed_step_solve, ode_count, ode_reinit, ode_fun, ode_solve, ode_jacobian, and ode_abs_tolerance. The use of macros improves readability because it hides the magic numbers used to indicate command type and because it hides dummy values used for parameters that are not needed by a specific command.

To permit rapid sharing of data between the NEURON core and Python extensions, we turned to a solution similar to that used for sharing of data between HOC Vector objects and NumPy arrays, turning a ctypes pointer into a NumPy array. Whichever function created the array does not regain control until after the handler completes. In this way, there is no risk of the memory being prematurely freed, so that the handler can safely manipulate this temporary array.

#### Analysis tools

The Model View tool, which provides a text and graphical summary of an instantiated model (Hines et al., 2007), was NEURON's first pre-existing analysis tool to be augmented with special support for reaction-diffusion. Like the other graphics and analysis tools, Model View already had access to concepts that overlapped with traditional NEURON, such as the presence or absence of calcium in a section.

We wrote code to add information about rxd models (e.g., Region, States, and Reaction) to the Model View GUI, but instead of integrating this code with the existing GUI code base, we left it in the rxd module. We then instantiate a _ModelViewExtension HOC template which we fill with pointers to the Model View updating code. The GUI now uses NEURON's List class to iterate over all _ModelViewExtension objects to have them add their data to the display. The immediate advantage of this architecture is that it removes the need for Model View to check if Python is available or if the rxd module has been imported. The long-term advantage is that it will allow future enhancements to add themselves to Model View with little or no changes to the existing code.

## Validation

### Analytic

**Figure 6:**
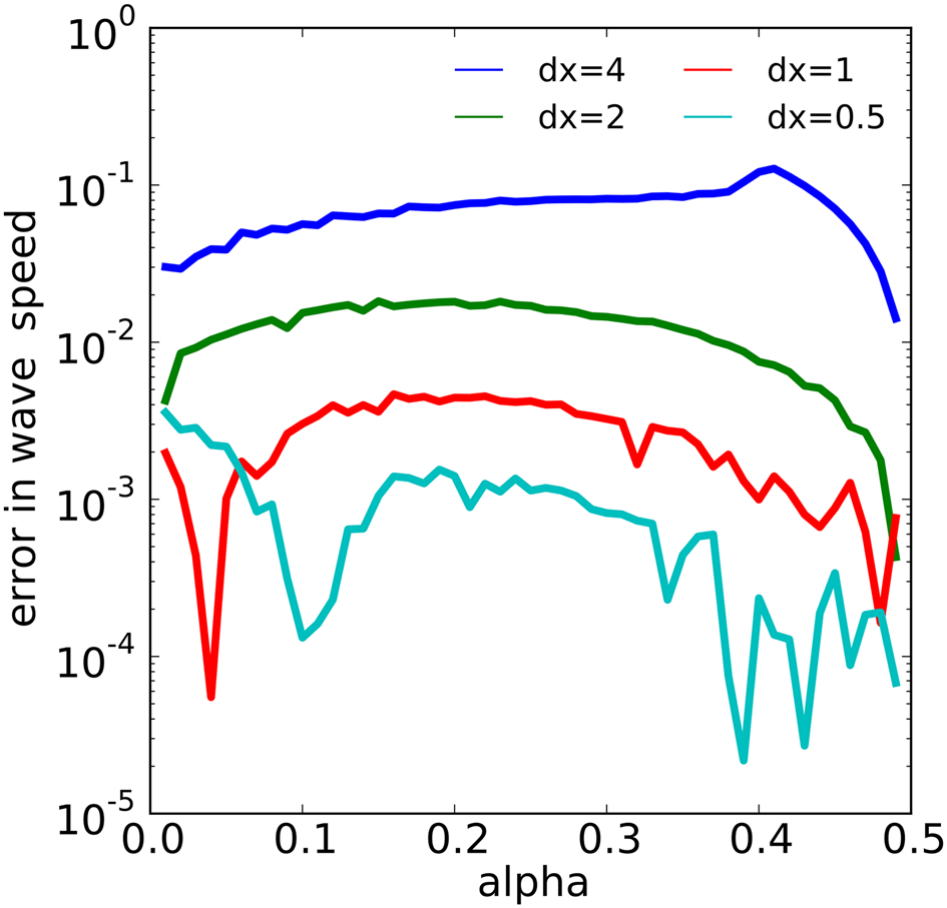
**The relationship between α and spatial discretization *dx* with the error in the computed wave speed in the model of example 1**. The error reduces by a factor of about 4 each time *dx* is halved over most of the range of α values.

The wave dynamics of example 1 have been proven to admit a traveling wave solution with velocity $c=\sqrt{2}\left(\frac{1}{2}-\alpha \right)$ on the infinite real line [see, for example, (Fife, 1979)]. We estimated the numerical wave speed as the average rate of speed of the wave measured from the threshold value α between *t* = 200 and *t* = 600. We then examined the error in the numerical wave speed for 49 different values of α evenly spaced in the interval [0, 0.49] for four different values of spatial discretization *dx* using NEURON's variable step solver with an absolute error tolerance of 10^−13^. We found that the numerical wave speed converged to the analytic wave speed approximately quadratically in *dx*, as shown in Figure 6. For example, for α = 0.25, with *dx* = 4, 2, 1, 0.5, we found numerical errors of about 0.07904, 0.01705, 0.004218, and 0.001136, respectively. The error behavior became more erratic as *dx* was reduced, especially near the extreme values of α.

### Numerical

Example 2 offers a more complex test for the reaction-diffusion extension, as electrical properties are now involved. In this case, there is no known analytic solution, so we compare to another numerical simulation. Fortunately, we can compare with the source for this example, (Fleidervish et al., 2010) which used NMODL to specify the same dynamics. Fleidervish et al. examined the sodium concentration over time at four locations in the neuron: the center of the soma, 40% of the way into the axon initial segment (AIS), 80% into the AIS, and 20% into the section they called myelin[0]. We compared the concentrations between the two versions of example 2 at *t* = 1500 ms simulated with NEURON's variable step solver with a relative tolerance of 10^−4^ and found the discrepancy between the two simulations was within 0.006% at all measured points.

## Discussion

Computational neuroscience has long been focused almost entirely on electrical phenomenology, mapping the neuron onto a resistor-capacitor circuit, and simulating using the electrical engineering approach pioneered by the SPICE simulator, followed by more specialized simulators such as GENESIS and NEURON. The coming of massive new datasets based on genome and proteome data focused more attention on the enormous complexity of molecular phenomenology, leading to the rise of computational systems biology to deal with these data in the various cell types of the body. Somewhat belatedly, this led to the realization that neurons are cells too, and that systems biology needed to be folded into computational neuroscience (De Schutter, 2008). In fact, neuronal molecular processing will likely feature greater complexity than seen in other cells, insofar as this molecular machinery must, among others, mediate the connection between the two major information stores in the brain: synapses and the genome/transcriptome. Synaptic plasticity, thought to underlie memory and learning, depends on local synaptic activations that signal changes in transcription to make molecular modification back at the synapse.

In many simulation domains, one can utilize a single approach, typically finite element or finite volume, to solve the entire problem. In the neuron, as in other cells, we find that many different problems coexist and must be solved simultaneously—the situation of multimodel or multiphysics. The level of detail for molecular simulation ranges from the high detail provided by simulators that track individual molecules, through stochastic simulators that use compartmentalization, up to deterministic diffusion simulators. In addition to different domains in the neuron requiring different solutions, different phases of information processing (e.g., during spiking or during synaptic activation) also require different solutions. This requirement has several implications. First we must define and deploy different solvers and different types of solutions depending on the context of part of the problem. Second, we must link across these different methods, methods which represent chemical entities in different ways. Finally, we must be able to define models at a sufficient level of abstraction so as to allow any of these different approaches to be swapped in and out as needed. This latter requirement largely dictated the approach to model definition described here, allowing the tool to define key model aspects, while remaining agnostic as to what type of simulation would be used to provide the solution.

Future extensibility and flexibility were key design goals. In this respect, use of Python offered several distinct advantages (Cornelis et al., 2012). The flexible syntax allowed us to specify the various aspects of the reaction-diffusion system readily in one language. This contrasted with NEURON's previous paradigm which used an interpreted language (HOC) to control and instrument the compiled code in NMODL. Python also allows our extension to directly utilize functions from the collection of scientific libraries, notably NumPy (numpy.org) and SciPy [scipy.org; Jones et al. (2001)], as well as external libraries accessible via Python. We use these libraries instead of writing custom code whenever possible because these libraries are likely to be low in bugs, and speed and space optimized. In addition, Python simplified development by automatically handling memory management, thereby reducing the risk of memory leaks and segmentation faults.

We extensively used Python's support for objects to allow for future extensibility by both users and developers. In our specification for reaction kinetics, rates are described as functions of the state variables. Operator overloading allows us to use the Python parser instead of a custom parser to process the rate formulas into an internal data structure that rxd later transparently converts into vectorized byte code. Any function that accepts and returns the same type of data structure may also be used in rate expressions. The rxd.math module contains analogs of the functions from math, and users may freely create and distribute more such functions. Likewise, for reaction specification, we maintain a list of instantiated objects that implement the necessary internal interface. If our reaction scheme proves inappropriate for some part of some model, the user may write those dynamics in a new class of their own construction without changing any of the rest of the system.

### Modularity

The approach taken here was meant to provide modularity not only for the internal architecture, but also for the choice of what reactions to represent at particular locations. Modularity increases extensibility by making it easier to mix-and-match pieces from different sources. At the level of diffusion modeling, the concept of a Region definition immediately provides the modularity required to allow one area to be simulated by one of a variety of stochastic simulators, such as Gillespie diffusion (Gillespie, 1976), Gillespie tau-leap (Gillespie, 2001), MCell (Stiles and Bartol, 2001), etc. Future enhancements will also involve dynamical adjustments to diffusive solutions such as adaptive grids. A more difficult enhancement would provide for dynamic reassignment of a region, as to whether requiring stochastic simulation or being adequately served by the more rapid deterministic solution.

We have tried to provide a generalized concept of Reaction. We necessarily provide a reaction-specification language for the direct entry of reaction schemes taken from the literature. However, it is expected that most reaction schemes will be taken from other modeling sources. This type of borrowing, encouraged throughout systems biology and computational neuroscience, is particularly useful for reaction schemes, which may have enormous complexity, involving hundreds of chemical species. In some scenarios, such as when the number of molecules of one or more species is low in one or more compartments, stochastic simulation may be more appropriate than deterministic simulation. Although, NEURON 7.3 does not support stochastic integration, we designed the Reaction specification to be independent of the integration type, so that in the future we can add support for stochastic integration without requiring changes to the model specification.

Alternatively, we might have used SBML (Hucka et al., 2003) as the native format for reaction specification. Indeed, SBML has established itself as the de facto standard for the exchange of models between systems biology simulators, and we are working on adding support for importing SBML into rxd. Nonetheless, we rejected SBML as a native format for rxd because the model domains are different: in NEURON, reaction-diffusion kinetics must interact with existing built-in and user-created ion channels and pumps distributed across spatially extended neurons and networks of neurons. SBML, however, does not have a concept of spatially extended models and any mapping to existing NEURON state variables must be done manually. NEURON models can introduce new dynamics mid-simulation; SBML generally describes a fixed set of dynamics. In principle, SBML Level 3's support for packages allows it to be infinitely extendable to address these and other short-comings, however models with dependencies on non-required extensions to the SBML core lose much of the portability that SBML aims to provide. Furthermore, SBML is verbose and difficult for humans to write. Finally, we note that since we used a plug-in based architecture for reaction specification, future tools for importing SBML, NeuroML (Gleeson et al., 2010), or VCML may internally express reactions in a completely different way, without requiring any changes to the existing rxd code.

An additional complexity for reactions in biological tissue is the distinction between a reaction within a compartment versus those that involve an intervening membrane. In the context of a neuron, this intervening membrane may or may not be excitable. If excitable, this requires additional coupling to active elements in the membrane which not only alter membrane voltage but also source or sink chemical species that are involved in reaction schemes. In order to best handle these coupling complexities, we exposed a number of previously internal NEURON functionalities as a new new nonvint_block_supervisor, providing a centralized place to add any new set of equations that can interact with other aspects of simulation and other solvers. Although currently only being used for rxd, this will allow other classes of dynamics to be simulated simultaneously with multiple solvers in a unified way.

### Implementation choices

Any implementation which both involves user programming and substantial numerics much strike the proper balance between interpreted (here Python) and compiled components. The interpreted language is too slow for the numerics, while the compiled language is too difficult for the user. This difficulty was here partially addressed by using the NumPy library. NumPy accepts vectorized specifications, and then performs the numerics using an underlying optimized C library. We could then reuse existing C code from NEURON, such as the treesolver algorithm, by passing in these NumPy vectors.

We anticipate that additional speed bottlenecks will arise in the future which may not be so readily solved by this expedient. We therefore have also begun to incorporate Cython, a variant of Python that translates Python code to C and then compiles it, into our development version, NEURON 7.4. With Cython, we can specify data types for key variables to eliminate the performance overhead which results from Python's loose typing methods. For classes, we can specify the full set of instance states in advance, to remove the overhead of hash table lookups. Cython lets us accelerate NumPy array lookups by skipping the call to __getitem__ and doing direct indexing. It also provides an additional way to integrate C code. To gain these performance benefits, some code modification is required. However, with few exceptions, such as issues with object properties, valid Python is valid Cython, allowing piece-by-piece transitioning without major rewrites.

### Alternative approaches

Multiple simulators in the computational neuroscience domain are adopting Python as a lingua franca so as to permit and encourage these programs to be used together. These connectors can then be used for a variety of purposes: data entry, data analysis, visualization, and co-simulation. Co-simulation is an alternative strategy for providing reaction-diffusion capability for NEURON, through coupling with an established simulator such as Vcell, MCell, NeuroRD, MOOSE, or STEPS. NEURON supports MUSIC, a framework for connecting simulators that has been used for both electrophysiology (Brocke and Djurfeldt, 2011) and reaction-diffusion models (Brandi et al., 2011).

While coupling of programs for data analysis or visualization can be reliably implemented, coupling for simulation is non-trivial for both conceptual and numerical reasons. Conceptually, the temporal or spatial discretizations may not be directly compatible. For example, NEURON traditionally uses arbitrarily-sized, arbitrarily-branching 1D sections in a tree morphology, while reaction-diffusion simulators use a grid—some using a cubic Cartesian grid while others use a tetrahedral grid. Technically, numerical artifacts and convergence failure can arise from weak coupling and from discrepancies across separate coupled simulators (Wils and De Schutter, 2009). In placing the reaction-diffusion simulator directly into NEURON, we still had to face the same issues but had the ability to change simulation technology on both sides as needed to make the fits work.

Having done this, we can now use NEURON with rxd as a reference tool with known numerical precision tolerance that will allow us to study coupling issues in the future (Cannon et al., 2007). The single-simulator and multi-simulator approaches offer important complementarity in terms of cross-verification. The single simulator approach used here allows the combination of all the disparate parts of the simulation into a single integrator, thereby directly confronting problems of stiffness and numerical stability that may be difficult to ferret out when dealing with different integrators exchanging data at fixed times. There are multiple other points of comparison, advantages and disadvantages to co-simulation using multiple small, single-purpose neural simulators versus larger all-in-one neural simulators, which can be explored further in the future (Ray and Bhalla, 2008).

Parallel with the technical discussion of coupling multiple simulators, multiscale modeling presents a series of conceptual choices between direct embedding and emergence embedding. In direct embedding, which has been discussed here, a lower-scale model is fully instantiated within the context of the higher-scale model. This contrasts with emergence embedding, where the emergent properties (the results) of the lower-scale model are captured and then included in a lumped fashion in the higher-scale model. These approaches are not mutually exclusive. Various degrees of simplification of the lower-scale model are frequently used to provide a lumped model that is believed to capture key emergent properties to a greater or lesser extent: for example the use of an integrate-and-fire model as proxy for a full Hodgkin–Huxley model, or the use of a mean-field approximation as a stand-in for a neuronal network. Both approaches are important, and our objective is to allow both strategies (Lytton and Hines, 2004; Lytton and Stewart, 2006; Kerr et al., 2013).

### Conflict of interest statement

The authors declare that the research was conducted in the absence of any commercial or financial relationships that could be construed as a potential conflict of interest.
